# Clinical Utility of the TRENDS Remote Monitoring Function Integrated into a Wearable Cardioverter-Defibrillator

**DOI:** 10.3390/s26123952

**Published:** 2026-06-22

**Authors:** Yoshifumi Ikeda, Risa Kanai, Yoshitaka Terazaki, Hitoshi Mori, Kazuhisa Matsumoto, Masataka Narita, Wataru Sasaki, Tsukasa Naganuma, Naomichi Tanaka, Ritsushi Kato

**Affiliations:** 1 Department of Cardiology, Saitama Medical University International Medical Center, 1397-1 Yamane, Hidaka 350-1298, Saitama, Japan; zin_ndmc@saitama-med.ac.jp (H.M.); m_kazu0@saitama-med.ac.jp (K.M.); n_masa19@saitama-med.ac.jp (M.N.); wata1107@saitama-med.ac.jp (W.S.); t_naganuma0518@saitama-med.ac.jp (T.N.); ntanaka@saitama-med.ac.jp (N.T.); ritsn@saitama-med.ac.jp (R.K.); 2 Department of Nursing, Saitama Medical University International Medical Center, 1397-1 Yamane, Hidaka 350-1298, Saitama, Japan; ri_kanai@saitama-med.ac.jp (R.K.); terazaki@saitama-med.ac.jp (Y.T.)

**Keywords:** wearable cardioverter-defibrillator, remote monitoring system, heart failure, implantable cardioverter-defibrillator, sudden cardiac death, compliance, arrhythmia, risk stratification

## Abstract

**Highlights:**

This study evaluated the clinical utility of TRENDS, a remote monitoring system integrated into a wearable cardioverter-defibrillator (WCD), in 36 prospectively enrolled patients and compared the findings with those of a historical control cohort. Daily WCD wear time was comparable between the TRENDS and historical control cohorts, whereas TRENDS provided additional physiologic and patient-reported information through remote monitoring. TRENDS data contributed to clinically important interventions, including optimization of heart failure therapy, detection of atrial fibrillation, initiation of anticoagulation therapy, and identification of reduced physical activity associated with indications for implantable cardioverter-defibrillator (ICD) implantation. These findings suggest that WCD-integrated remote monitoring may support not only arrhythmia surveillance but also early risk stratification and individualized cardiovascular management during the vulnerable post-discharge period.

**What are the main findings?**

- Daily WCD wear time was comparable between the TRENDS and historical control cohorts, while TRENDS provided additional physiologic and patient-reported information through remote monitoring.
- TRENDS-derived physiologic and symptom data facilitated early detection of clinical deterioration and supported individualized therapeutic interventions.

**What are the implications of the main findings?**

- WCD-integrated remote monitoring may broaden the role of WCDs from temporary arrhythmia protection to comprehensive cardiovascular management.
- Continuous monitoring of physiologic and behavioral parameters may enable earlier intervention during vulnerable phases of heart failure and arrhythmia care.

**Abstract:**

Background: Wearable cardioverter-defibrillators (WCDs) are equipped with the TRENDS remote-monitoring system, enabling continuous assessment of arrhythmias, physiological parameters, and patient-reported outcomes. This study evaluated the clinical utility of TRENDS-integrated WCD management and compared it with a historical control. Methods: We prospectively analyzed 36 consecutive patients who received a WCD with TRENDS between 2019 and 2024 and compared them with 30 historical controls treated before the implementation of TRENDS. Results: The WCD indications were heart failure as primary prevention (64%) and acute coronary syndrome with ventricular arrhythmias (28%). Among 18 patients who met the criteria for an implantable cardioverter-defibrillator (ICD), including 1 patient with WCD shock, 9 ultimately underwent ICD implantation. The mean daily WCD wear-time was 21.3 h and did not differ significantly from that of the historical control. The response rate to health-related questionnaires was 89%. TRENDS detected symptom exacerbation in 31% of patients, weight gain in 19% of patients, and missed medication in 19% of patients. Daily step-count was significantly lower in patients with ICD indications than in those without (5012 ± 2980 steps vs. 7977 ± 3584 steps, *p* = 0.01). TRENDS data also aided in initiating anticoagulation therapy and optimizing beta-blocker therapy. Conclusions: TRENDS provided clinically actionable physiologic and patient-reported information that supported individualized cardiovascular management.

## 1. Introduction

Wearable cardioverter-defibrillators (WCDs) are non-invasive devices primarily indicated for the prevention of sudden cardiac death in patients with a transiently elevated risk who are not yet candidates for implantable cardioverter-defibrillators (ICDs) [1]. WCDs are most commonly prescribed in the early phase after acute coronary syndrome, in newly diagnosed heart failure (HF) with reduced ejection fraction, or perioperatively during ICD removal [2]. While the primary function of ICDs is arrhythmia detection and shock delivery, emerging evidence suggests that WCDs may serve a broader role in patient management, particularly when integrated with remote monitoring systems (RMSs) [3]. Previously, an RMS attached to a WCD could be used only to monitor arrhythmia events, but the TRENDS (ZOLL Medical Corporation; https://cardiac.zoll.com/) RMS and health-related questionnaire were added in 2019. This novel RMS enables observation of not only arrhythmic events but also physiologic parameters, such as step count, heart rate, and body angle, as well as the patient’s responses to health-related questionnaires. When integrated with a WCD, an RMS can provide real-time data to clinicians, potentially allowing for earlier detection of clinical deterioration, optimization of pharmacologic therapy, and improved patient management [4,5]. However, while adoption of WCDs in cardiac care remains high, clinical data are limited regarding the impact of WCD use when combined with an RMS in routine practice [6].

This study aimed to evaluate the potential clinical utility of an RMS-integrated WCD in patients with various cardiac indications. Specifically, we investigated how physiologic and behavioral metrics collected during WCD use may inform therapeutic decision-making, adherence monitoring, and early intervention in the management of HF and arrhythmias.

## 2. Materials and Methods

### 2.1. Study Design and Population

This observational cohort study consisted of a prospectively enrolled TRENDS cohort and a retrospectively identified historical control cohort.

The TRENDS cohort included 36 consecutive patients who were prescribed a WCD between 2019 and 2024 at Saitama Medical University International Medical Center following implementation of the TRENDS RMS.

The historical control cohort consisted of 30 consecutive patients who received WCD therapy at the same institution before implementation of the TRENDS system and were managed according to standard WCD care without access to TRENDS-derived remote monitoring data.

Patients were enrolled on the basis of clinical indications for WCD use as determined by a board-certified cardiologist, in accordance with the Japanese Circulation Society/Japanese Heart Rhythm Society 2019 Guideline on Non-Pharmacotherapy of Cardiac Arrhythmias [7].

### 2.2. Inclusion Criteria

Eligible patients were required to meet one or more of the following guideline-based criteria and be available for evaluation using remote monitoring via the LifeVest Network (ZOLL Medical Corporation, Pittsburgh, PA, USA):

1. Left ventricular ejection fraction (LVEF) ≤ 35% within 40 days following acute myocardial infarction, with New York Heart Association (NYHA) class II–III symptoms.
2. LVEF ≤ 35% within 90 days after coronary artery bypass grafting or percutaneous coronary intervention, with NYHA class II–III symptoms.
3. LVEF ≤ 35% due to non-ischemic acute HF within 90 days of onset, with NYHA class II–III symptoms.
4. End-stage HF meeting the listing criteria for heart transplantation.
5. Patients for whom ICD implantation was indicated but could not be immediately performed because of other physical conditions.
6. Patients who underwent temporary extraction of an ICD because of infection or other causes.
7. Patients considered for ICD-based sudden cardiac death prevention but in whom a period of observation was prioritized.
8. Hospitalized patients at moderate risk of life-threatening arrhythmias for whom continuous monitoring was not feasible.

### 2.3. Exclusion Criteria

Patients were excluded if they met any of the following conditions:

1. The patient was deemed not to require WCD prescription on the basis of clinical judgment;
2. Data could not be reliably acquired from the LifeVest Network (because of an extremely short duration of wear, communication issues, or device malfunction);
3. The patient exercised the right to opt out of the study.

### 2.4. Data Collection and Endpoints

Baseline demographic and clinical data, namely, age, sex, body surface area, annual income, comorbidities, medical history, vital signs, laboratory values, and medication profiles, were collected at the time of WCD initiation.

The observation period was limited by the Japanese medical insurance system, with coverage starting from the month of hospital discharge (considered month 1) and extending to a maximum of 3 months. Therefore, the duration of WCD wear varied between patients depending on their discharge date.

The primary endpoints comprised:

- Major cardiac events (e.g., coronary artery disease, life-threatening arrhythmias, and HF hospitalizations) during WCD use;
- ICD implantation during the observation period;
- Changes in medication regimens;
- Other invasive cardiac interventions.

All enrolled patients were included in the analyses of clinical outcomes and WCD-related events. However, patients who discontinued WCD use within the first month and lacked sufficient follow-up data were excluded only from longitudinal analyses of echocardiographic parameters, laboratory measurements, and medication changes.

In addition, baseline characteristics, WCD adherence, clinical outcomes, and device-related parameters were compared between the TRENDS cohort and the historical control cohort to evaluate the potential clinical utility of TRENDS-guided monitoring.

### 2.5. Remote Monitoring via the LifeVest Network

WCD data were transmitted daily, or after any shock event, from the wearable device to a docking station (charger), which functioned as a Bluetooth-enabled transmitter. The information was subsequently uploaded via a mobile network to the LifeVest Network, which is a cloud-based remote monitoring system managed by ZOLL Medical Corporation. This system (TRENDS) provided access to defibrillation data, arrhythmia episodes, heart rate trends, daily step counts, wear time, and body posture metrics indicative of heart failure status.

**Figure 1:**
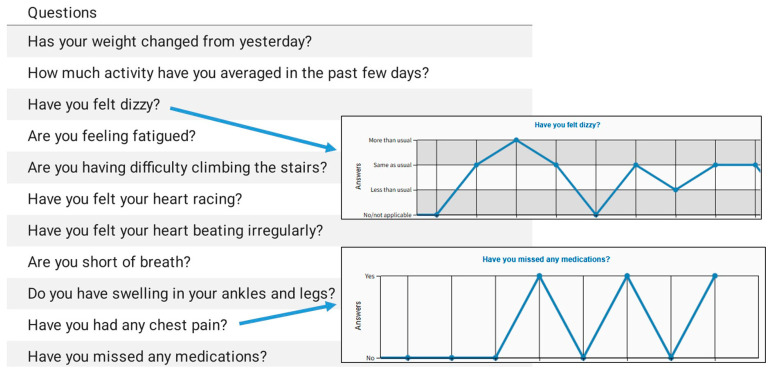
Weekly health-related questionnaire. Patients were requested to complete the questionnaire once per week. The patients’ responses were accessible to healthcare professionals via the RMS. For example, questions assessed patient reactions to dizziness and missed medication doses.

The WCD interface also allowed patients to respond to the health-related questionnaire displayed on the device. The health-related questionnaire is shown in Figure 1, and example items include questions related to patient reactions to dizziness and missed medication doses. The health-related questionnaire function was available only in the TRENDS cohort. Consequently, questionnaire adherence could not be directly compared with the historical control cohort.

**Figure 2:**
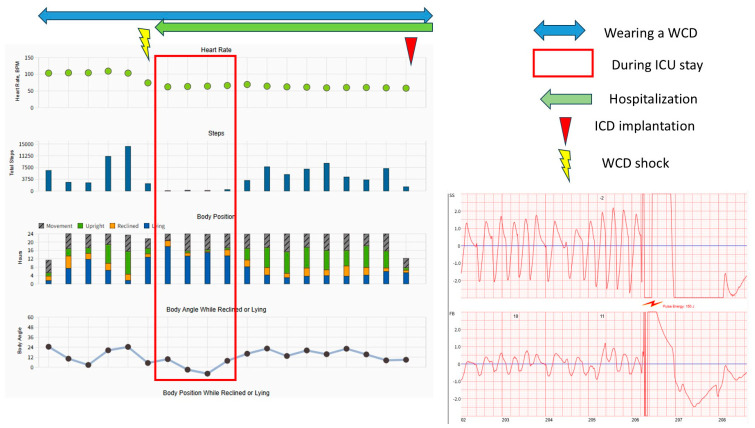
In Case 30, the patient’s clinical course aligned with the TRENDS display and WCD shock event. From top to bottom, the TRENDS display shows heart rate, step count, body position (movement, upright, reclined, and lying down), and body angle. Additional symbols are as follows: yellow lightning bolt, WCD shock; red inverted triangle, ICD implantation; red square, ICU stay; blue arrow, WCD wear period; and green arrow, hospitalization period. The bottom-right panel shows the ECG recorded by the WCD at the time of the shock. Abbreviations: ICD, implantable cardioverter-defibrillator; ICU, intensive care unit; ECG, electrocardiogram; WCD, wearable cardioverter-defibrillator; bpm, beats per minute.

Prior to discharge, patients were trained to complete the health-related questionnaire as part of their HF education and were requested to answer the questionnaire once weekly. At our center, two dedicated nurses with ICD training reviewed these data and managed alerts. An example of TRENDS data from Case 30 is shown in Figure 2.

This patient experienced worsening heart failure shortly after discharge, received an appropriate WCD shock, and was subsequently readmitted for ICD implantation. The observed increase in the proportion of upright posture events and increased body angle prior to WCD activation suggested worsening congestion, while reduced step counts indicated restricted mobility in the intensive care unit following readmission.

### 2.6. Statistical Analysis

The normality of continuous variables was assessed using the Shapiro–Wilk test. Continuous variables with normal distributions were compared using Student’s *t*-test, while non-normally distributed variables were analyzed using the Mann–Whitney U test. Categorical variables were compared using the chi-squared (χ^2^) test. Comparisons were performed both within the TRENDS cohort and between the TRENDS cohort and the historical control cohort, as appropriate. A two-tailed *p*-value < 0.05 was considered statistically significant. All statistical analyses were performed using SPSS Statistics version 21.0 (IBM Corp., Armonk, NY, USA).

Because this study was designed as an exploratory observational study to evaluate the clinical utility of TRENDS-derived information in routine clinical practice, a formal a priori sample size calculation was not performed. Accordingly, the results should be interpreted as hypothesis-generating.

## 3. Results

The patients’ characteristics are summarized in Table 1.

The baseline demographic and clinical characteristics were generally comparable between the TRENDS cohort (*n* = 36) and the historical control cohort (*n* = 30). There were no significant differences in age, sex, NYHA class, body size, etiology of cardiomyopathy, or major comorbidities between the two groups.

With respect to WCD indications, the proportion of patients classified as having “other” indications was significantly lower in the TRENDS cohort than in the historical control cohort (8% vs. 33%, *p* = 0.001). No significant differences were observed in the proportions of patients receiving WCDs for primary prevention, secondary prevention, acute coronary syndrome, or heart failure. The cohort in the present study was relatively young, with a mean age of 58.5 ± 12.7 years, and the majority of the patients were men (86%, *n* = 31). Ischemic heart disease was present in 39% (*n* = 14) of the patients, and 72% (*n* = 28) were indicated for WCD as primary prevention. The mean duration of WCD use was 61.7 ± 21.8 days, with a mean daily wear time of 21.3 ± 4.9 h. The mean duration of WCD use was similar between the TRENDS and historical control cohorts (61.7 ± 21.8 vs. 59.9 ± 27.8 days, *p* = 0.78). Likewise, the mean daily wear time did not differ significantly between groups (21.3 ± 4.9 vs. 20.0 ± 5.7 h, *p* = 0.36). The response rate to the weekly health-related questionnaires was 89%, indicating good overall compliance. Because the health-related questionnaire function was not available in the historical control cohort, direct comparison of questionnaire adherence was not possible. Arrhythmias, including both atrial and ventricular arrhythmias, occurred in 69% (*n* = 25) of the patients. Manual termination of shock therapy was performed in 8% (*n* = 3) of the patients. No inappropriate shocks were observed, and appropriate therapy was delivered in a single case. Clinical outcomes, including heart failure hospitalization, ICD indication, and ICD implantation, were not significantly different between the TRENDS and historical control cohorts. Of the total cohort, 50% (*n* = 18) were deemed eligible for ICD implantation; however, only 25% (*n* = 9) ultimately underwent device implantation. In one patient (Case 20), newly diagnosed atrial fibrillation detected via remote monitoring led to the initiation of a direct oral anticoagulant, while in another patient (Case 25) with a persistently elevated heart rate, the addition of a beta-blocker was recommended. Table 2 summarizes the examination findings and their changes during the study period.

Two patients discontinued WCD use within the first month, one because of dermatological adverse events and the other because of early ICD implantation following an appropriate WCD shock. These patients remained included in the overall study population and outcome analyses; however, they were excluded from longitudinal analyses of echocardiographic, laboratory, and medication-related changes because complete follow-up data were unavailable. The patients’ mean heart rate decreased significantly from 90.0 ± 35.8 to 67.5 ± 11.9 bpm (*p* = 0.03). LVEF increased significantly from 33.5% ± 16.8% to 40.1% ± 13.7%, and LV dimensions decreased markedly (end-diastolic/systolic diameters: from 60.2 ± 9.8/49.5 ± 12.6 mm to 55.3 ± 9.8/42.5 ± 10.5 mm; *p* = 0.003/<0.001). No other significant changes were observed in the laboratory parameters. In the historical control cohort, the mean heart rate also decreased significantly during follow-up; however, no significant changes were observed in LVEF, LV dimensions, or laboratory parameters.

Data on medication use are also presented in Table 2. Among the 36 patients, medications were adjusted in 34 individuals, excluding 2 patients who discontinued WCD use within 1 month because of dermatological adverse events and early ICD implantation following a WCD shock, respectively. The prescription rates of guideline-directed medical therapy for HF increased as follows: from 61% to 88% for angiotensin-converting enzyme inhibitors/angiotensin receptor blockers/angiotensin receptor-neprilysin inhibitors; from 33% to 45% for beta-blockers; and from 58% to 67% for mineralocorticoid receptor antagonists. Medication adjustments were also performed in the historical control cohort; however, despite optimization of medical therapy, significant improvements in LVEF and LV remodeling were not observed.

**Figure 3:**
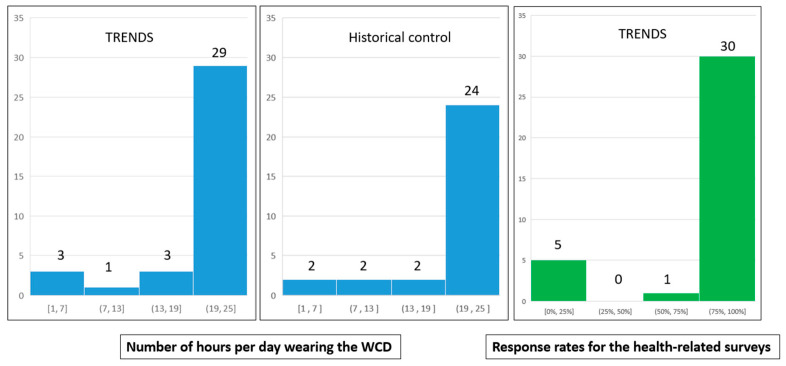
Number of hours per day of WCD wear and response rates to health-related questionnaires. The left panel shows the wear time in the TRENDS cohort, the middle panel shows the wear time in the historical control cohort, and the right panel shows the response rates to the weekly health-related questionnaires in the TRENDS cohort. The health-related questionnaire function was not available in the historical control cohort. Abbreviations: WCD, wearable cardioverter-defibrillator.

Figure 3 shows the distributions of daily WCD wear time in the TRENDS and historical control cohorts, as well as the response rates to the weekly health-related questionnaires in the TRENDS cohort. In the TRENDS cohort, 29 of 36 patients (81%) wore the WCD for at least 19 h per day. Similarly, in the historical control cohort, 24 of 30 patients (80%) wore the WCD for at least 19 h per day, indicating comparable adherence between the two groups. In the TRENDS cohort, 30 of 36 patients (83%) responded to at least 75% of the health-related questionnaires. Because the questionnaire function was not available in the historical control cohort, no comparison of questionnaire adherence could be performed.

**Figure 4:**
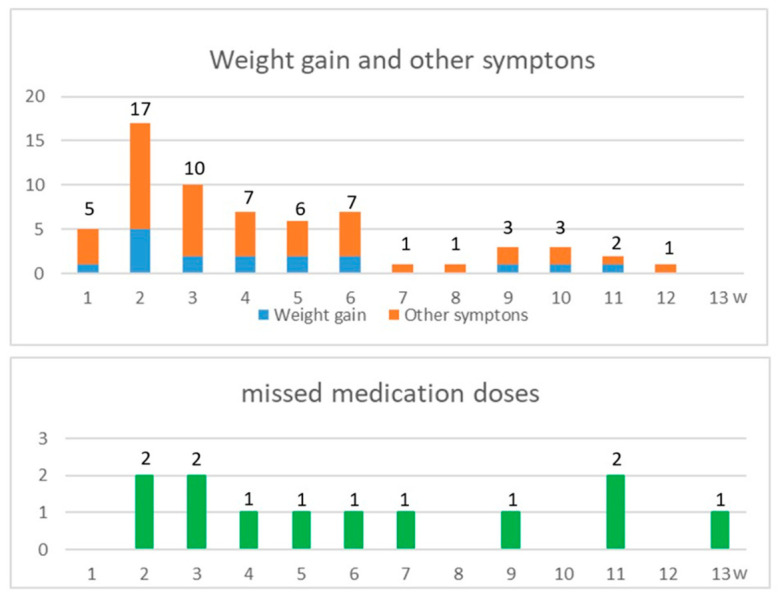
Occurrence of symptoms, weight gain, and missed medication doses during the WCD wear period observed in the TRENDS RMS data. The orange bars indicate the number of reported symptoms; blue bars indicate the number of weight gain events; and green bars indicate the number of missed medication doses. Abbreviations: WCD, wearable cardioverter-defibrillator; RMS, remote monitoring system; w, weeks.

Figure 4 illustrates the weeks during which symptoms, weight gain, and missed medication doses occurred during WCD use, covering a maximum of 13 weeks. No specific trend was observed in missed medication doses; however, weight gain and other symptoms occurred primarily within the first 6 weeks, with >60% occurring within 4 weeks and with a peak in the second week. Changes in body angle suggestive of orthopnea were observed in 13% (*n* = 5) of the patients, two of whom were subsequently hospitalized for HF.

**Figure 5:**
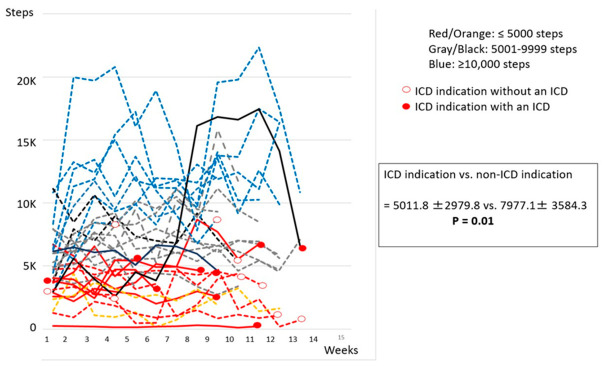
Step count trends obtained from the TRENDS feature. The solid lines represent patients with ICDs; dotted lines represent those without. Red and orange indicate average step counts <5000; black and gray, 5001–9999 steps; and blue, ≥10,000 steps. Orange and gray represent patients without ICD indications. Red circles denote ICD indications; filled circles, ICD implantation; and open circles, no ICD implantation. The red lines represent patients with mean step counts ≤5000 who met the criteria for ICD implantation. The solid lines indicate patients who underwent ICD implantation, whereas the dotted lines indicate those who did not. The orange dotted lines represent patients with ≤5000 steps who did not meet the ICD criteria. The black lines represent patients with step counts between 5001 and 9999 who met the ICD criteria; solid and dotted lines distinguish ICD recipients and non-recipients, respectively. The gray dotted lines indicate patients within this step range who were not eligible for ICD implantation. Abbreviations: ICD, implantable cardioverter-defibrillator; K, thousand.

Figure 5 illustrates the changes in physical activity over time based on step counts. Notably, there was only one ICD-indicated case with a mean step count ≥10,000, represented by the blue dotted line. Patients with definite ICD indications tended to cluster below 5000 steps. The mean step count was significantly lower in the ICD indication group than in the other groups (5011.8 ± 2979.9 vs. 7977.1 ± 3584.3 steps, respectively; *p* = 0.01).

## 4. Discussion

The key findings of the present study are as follows: First, patients who were ultimately judged to meet the ICD indication criteria had significantly lower step counts than those who were judged not to meet the criteria (5011.8 ± 2979.8 vs. 7977.1 ± 3584.3 steps, respectively; *p* = 0.01) on the basis of the TRENDS data, suggesting that reduced physical activity may reflect greater disease severity or advanced cardiac dysfunction.

Second, in this single-center prospective cohort of Japanese patients using a WCD integrated with an RMS, adherence to device use was favorable, with a mean daily wear time of 21.3 ± 4.9 h. Moreover, the response rate to the embedded weekly health-related questionnaires was high (89.0%), indicating that patients consistently engaged with the self-monitoring platform. However, comparison with a historical control cohort demonstrated no significant difference in daily WCD wear time, suggesting that favorable device adherence was not attributable solely to the TRENDS monitoring system.

Third, symptoms and weight gain reported through the health-related questionnaires were most frequently observed within the first 6 weeks after discharge, peaking near the second week. Notably, physiologic information derived from the TRENDS system contributed to several clinical decisions, including initiation of anticoagulation for newly detected atrial fibrillation and up-titration of β-blockers in response to an elevated heart rate. These findings suggest that TRENDS may provide clinically actionable information during routine patient management; however, owing to the observational study design, a causal effect on clinical outcomes cannot be established.

Collectively, these findings suggest that TRENDS-based remote monitoring may facilitate early risk stratification and support individualized management strategies during the early post-discharge phase of acute cardiac disease.

### 4.1. Effectiveness of the TRENDS Function

The TRENDS function provides continuous remote monitoring of heart rate, physical activity, arrhythmias, and body angle, and it routinely prompts patients to complete health-related questionnaires. Previous studies have demonstrated the utility of TRENDS in HF risk stratification and as an educational tool for promoting self-management [8]. Step count data obtained from WCDs have also been associated with the risk of ventricular tachycardia/ventricular fibrillation events, supporting WCD use as a noninvasive biomarker [9].

Although the number of arrhythmic events in our study was limited, the significantly lower activity levels observed in patients requiring versus not requiring ICD implantation suggest that reduced mobility may indicate a greater disease burden and poorer recovery potential. Accordingly, quantitative assessment of physical activity using pedometer-derived data from the TRENDS system may serve as a biomarker of impaired recovery of cardiac function. However, the present findings should not be interpreted as demonstrating prediction of ventricular arrhythmic events, as only one appropriate WCD shock occurred during follow-up. Rather, reduced physical activity may reflect overall disease severity and the likelihood of subsequently meeting ICD implantation criteria. Likewise, the improvements observed in LVEF and left ventricular dimensions during follow-up should not be attributed solely to the TRENDS monitoring system. Reverse remodeling during the WCD period is expected in patients recovering from acute heart failure, acute myocardial infarction, and newly diagnosed cardiomyopathies receiving guideline-directed medical therapy, and similar findings have been reported in previous WCD studies. Although TRENDS may have supported clinical decision-making and treatment optimization in selected cases, its independent contribution to cardiac functional recovery cannot be determined from the present observational study.

Furthermore, activity metrics derived from implanted devices have been reported to predict appropriate ICD therapies and HF hospitalizations, enabling continuous risk stratification even after ICD implantation [10]. In the present cohort, a visual clustering of patients with ICD indications at lower activity levels was observed. However, we did not intend to propose a specific step-count threshold for risk stratification, and the study was not designed to identify or validate an optimal cutoff value. Therefore, the observed association between reduced physical activity and ICD indication should be considered exploratory and hypothesis-generating. Larger studies are required to determine whether clinically meaningful activity thresholds can be established and validated for risk assessment.

In the present study, early detection of new-onset atrial fibrillation and elevated resting heart rates led to timely pharmacologic interventions. However, the weekly assessment of the health-related questionnaire may have delayed HF management, as evidenced by hospitalizations despite preceding physiologic changes. Increasing the frequency of assessments might enhance sensitivity to early decompensation.

### 4.2. Impact of WCD Compliance

A large French registry reported a mean daily WCD wear time of 23.4 h, although compliance tended to be lower among younger patients than among older patients [11]. In the VEST trial, which was a multicenter randomized controlled trial in post-myocardial infarction patients, compliance followed a U-shaped distribution [12]. While the intention-to-treat analysis failed to show a significant mortality reduction, both as-treated and per-protocol analyses demonstrated lower arrhythmic and non-arrhythmic mortality among patients with higher adherence.

Despite the recognized importance of WCD compliance, strategies to improve patient adherence remain uncertain. In the present study, adherence to WCD wear was favorable, with a mean daily wear time of 21.3 ± 4.9 h, which was comparable to that reported in previous registries. Importantly, a comparison with the historical control cohort revealed no significant difference in daily WCD wear time, suggesting that the favorable adherence observed in the present study cannot be attributed solely to the TRENDS monitoring system. A unique feature of the present study was the evaluation of patient engagement with the health-related questionnaire function integrated into TRENDS. The response rate was high throughout the observation period, indicating that patients were willing to participate in regular self-assessment while wearing the WCD. In addition, the questionnaire includes items related to missed medication doses, enabling the identification of self-reported medication nonadherence during the early phase of cardiovascular treatment. Unfortunately, the present study did not employ a validated measure of medication adherence, and, therefore, the impact of TRENDS on medication compliance could not be formally evaluated. Accordingly, our findings should not be interpreted as evidence that TRENDS improves medication adherence, but rather that it may provide additional information regarding patient-reported treatment behaviors that could support clinical decision-making.

Predischarge transition programs reduce readmissions, and indicators derived from patient-reported HF assessment tools may assist in prognostic evaluation [13]. Therefore, the value of TRENDS may lie not in improving WCD adherence itself but in providing additional patient-reported and physiologic information that can support clinical decision-making during the vulnerable post-discharge period. However, further studies are needed to determine whether structured education on health-related questionnaire use directly enhances WCD adherence and clinical outcomes.

### 4.3. Limitations of the TRENDS Feature

In the present study, symptom alerts recorded by the TRENDS function were concentrated within the first 6 weeks after discharge, particularly within the first 4 weeks, with a peak during the second week (Figure 4). Rehospitalization within 30 days after an HF admission is associated with a poor prognosis [14], and a previous study reported that approximately 65% of HF readmissions occur within 15 days of discharge [15]. Early post-discharge follow-up reduces the risk of rehospitalization, suggesting that continuous symptom monitoring using TRENDS could be a practical tool to mitigate early readmissions in HF patients [16].

Although the sample size was limited, arrhythmia detection, heart rate trends, and activity data obtained through the TRENDS RMS were useful for making medication adjustments. Nonetheless, these features were insufficient to prevent rehospitalization in two patients who experienced HF exacerbation in the present study. A key issue may be the frequency of the health-related questionnaires. In this study, this symptom evaluation was performed weekly, which may have been insufficient for detecting early deterioration during the vulnerable post-discharge phase, particularly in patients indicated for WCD use. Given that symptom alerts were concentrated within the first 6 weeks after discharge, with a peak during the second week, more intensive monitoring during this period may improve early identification of clinical deterioration. In particular, a flexible strategy incorporating twice-weekly health-related questionnaires during the early post-discharge phase may facilitate more timely clinical intervention. Future studies should evaluate whether increased questionnaire frequency and adaptive monitoring protocols can improve the sensitivity of TRENDS-guided management and reduce rehospitalization risk. However, the optimal frequency of patient-reported assessments remains unknown. While more frequent monitoring may facilitate earlier detection of clinical deterioration, excessive questionnaire burden could adversely affect patient engagement and long-term adherence. Future studies should therefore investigate the balance between monitoring intensity, patient burden, and clinical effectiveness. More frequent assessments—especially within the first 6 weeks—may be necessary to improve early detection of decompensation. Given the limitations of relying on single physiologic indicators, modern remote monitoring platforms have adopted multisensor algorithms to improve HF event prediction. The MultiSENSE study introduced the HeartLogic™ (Boston Scientific Corporation, Marlborough, MA, USA) index, which integrates multiple physiologic parameters, including heart sounds, activity, and thoracic impedance, to predict impending HF decompensation, achieving a median lead time of 37 days [17]. Similarly, HeartInsight (Biotronik, Berlin, Germany), which combines heart rate trends, arrhythmia burden, physical activity, and thoracic impedance, demonstrated high predictive accuracy, with positive and negative predictive values of 5.6–7.7% and 96.6–96.7%, respectively [18].

Although the TRENDS function of the WCD currently does not incorporate a multisensor algorithm, recent studies suggest that coupling WCDs with vibrometer-derived acoustic sensors may achieve comparable predictive performance [19].

However, direct comparisons between TRENDS and commercially available multisensor monitoring systems such as HeartLogic™ and HeartInsight should be interpreted with caution. These systems are integrated into implantable cardiac devices and generate composite risk scores based on multiple physiologic parameters and predefined alert thresholds, whereas TRENDS primarily provides individual physiologic measurements and patient-reported information without an integrated predictive algorithm.

Nevertheless, the clinical effectiveness of multisensor algorithms remains a subject of ongoing debate. Variability in performance, the potential for false-positive alerts, and the increased monitoring burden on clinicians represent important limitations [20]. In contrast to purely sensor-based approaches, TRENDS incorporates a health-related questionnaire that allows active patient participation in the monitoring process. This feature may provide additional clinically relevant information regarding symptoms, medication adherence, and perceived health status that cannot be captured by physiologic sensors alone. The combination of objective physiologic data and patient-reported outcomes may represent a unique advantage of WCD-based monitoring during the vulnerable post-discharge period. Rather than serving as a direct alternative to implantable multi-sensor algorithms, TRENDS may be viewed as a complementary monitoring platform designed for temporary use in high-risk patients during the early phase of cardiovascular disease management. Recent advances in wearable sensor technologies, cloud-based monitoring platforms, and remote biomedical infrastructures have substantially improved the reliability and scalability of continuous physiologic monitoring [21]. Modern RMSs are increasingly capable of integrating data acquisition, transmission, storage, and clinical review within a unified framework, enabling near real-time assessment of patient status outside the hospital setting. Such technological developments have facilitated the broader adoption of remote monitoring strategies in cardiovascular medicine and support the feasibility of systems such as TRENDS for longitudinal patient management.

In this context, the TRENDS system—which incorporates patient-reported symptom data—may help address some of the limitations of purely sensor-based approaches. To maximize its clinical utility, future development should focus on integrating physiologic measurements and patient-reported outcomes into composite risk scores and adaptive monitoring strategies. Recent advances in multisensor monitoring and artificial intelligence have demonstrated the potential of combining heterogeneous data sources to improve risk prediction and individualized clinical decision-making [22]. Because TRENDS already collects multiple physiologic and patient-reported parameters, incorporation of such approaches may further enhance early identification of high-risk patients and support more timely intervention. Another important consideration for RMSs with wearable devices is the reliability and tolerability of physiologic signal acquisition. Continuous ECG monitoring requires prolonged skin contact, which may affect both signal quality and patient adherence. In the present study, one patient discontinued WCD use because of dermatological adverse events, highlighting the importance of patient comfort during long-term monitoring. Recent advances in wearable ECG technologies have focused on improving sensor materials, skin compatibility, and signal stability while reducing motion artifacts and patient discomfort. Such developments may enhance both the quality of physiologic data acquisition and long-term adherence to wearable monitoring systems [23].

### 4.4. Study Limitations

This study has several limitations. First, this was a single-center observational study with a small sample size and a limited number of events, which reduced the statistical power. Because this study was designed as an exploratory observational investigation, a formal a priori sample size calculation was not performed. Therefore, the study may have been underpowered to detect modest but clinically meaningful differences, particularly for outcomes with low event rates. In addition, the limited sample size and number of patients meeting ICD indication criteria precluded reliable multivariable analyses adjusting for established clinical risk factors. Consequently, the observed association between reduced physical activity and ICD indication should be interpreted as exploratory and hypothesis-generating rather than evidence of an independent predictor. Although a historical control cohort was included for comparative analyses, the absence of randomization and the use of non-contemporaneous controls limit the ability to infer causality. Furthermore, residual confounding, selection bias, and temporal changes in clinical practice cannot be completely excluded.

In particular, the small sample size may have reduced the power to detect significant changes in laboratory parameters such as BNP and eGFR, both of which are known to exhibit substantial inter- and intra-individual variability. Therefore, the lack of statistical significance in these markers should be interpreted with caution. Similarly, nonsignificant findings in other exploratory analyses should not be interpreted as evidence of the absence of an association.

Additionally, there was no long-term follow-up beyond the WCD wear period.

Furthermore, two patients who discontinued device use within the first month were excluded from the primary analysis due to insufficient follow-up data. However, a sensitivity analysis including these patients demonstrated that the overall trends and main findings were not materially altered. Nevertheless, the potential impact of early discontinuation cannot be entirely excluded.

In addition, the health-related questionnaire function was available only in the TRENDS cohort. Therefore, a direct comparison of patient engagement and questionnaire adherence between the TRENDS and historical control cohorts was not possible.

Finally, given the observational design, the use of a historical control cohort, and the relatively small sample size, the present findings should be interpreted as descriptive and hypothesis-generating rather than confirmatory. Accordingly, a direct causal relationship between RMS use and the reduction of adverse events cannot be established.

Future multicenter studies with larger patient populations and adequate statistical power are warranted to validate the clinical utility of the TRENDS monitoring function and other remote metrics in guiding early intervention and improving clinical outcomes. Although randomized controlled trials would provide the highest level of evidence, large-scale prospective multicenter studies may also help clarify the clinical value of TRENDS-guided monitoring in routine practice.

## 5. Conclusions

Wearable cardioverter-defibrillators implemented early after the onset of cardiac disease offer valuable physiologic and behavioral insights through the integration of TRENDS RMS data. This integration with WCDs may facilitate earlier interventions for HF and arrhythmia risk, highlighting the potential role of these devices as both life-saving devices and comprehensive remote monitoring tools.

## Figures and Tables

**Table 1:** Patients’ characteristics and clinical outcomes.

Parameters	TRENDs *n* = 36	Historical Control *n* = 30	*p*-Value
Basic information			
Male	86% (*n* = 31)	73% (*n* = 22)	*0.19*
NYHA classification	2.6 ± 0.6	2.3 ± 0.7	*0.40*
Age (y)	58.5 ± 12.7	52.3 ± 16.8	*0.09*
Ht (cm)	164.8 ± 6.8	165.1 ± 8.9	*0.90*
Wt (kg)	67.9 ± 13.7	63.8 ± 15.4	*0.25*
BSA (m^2^)	1.7 ± 0.2	1.6 ± 0.4	*0.15*
Etiology			
IHD	39% (*n* = 14)	33% (*n* = 10)	*0.56*
NIHD	61% (*n* = 22)	67% (*n* = 20)	*0.85*
Indication of WCD			
Primary prevention	72% (*n* = 28)	57% (*n* = 17)	*0.06*
Secondary prevention	28% (*n* = 8)	43% (*n* = 13)	*0.06*
ACS	28% (*n* = 10)	17% (*n* = 5)	*0.31*
HF	64% (*n* = 23)	63% (*n* = 19)	*0.78*
Other	8% (*n* = 3)	33% (*n* = 10)	*0.001*
Comorbidity			
HT	42% (*n* = 15)	37% (*n* = 11)	*0.68*
HL	50% (*n* = 18)	37% (*n* = 11)	*0.28*
DM	36% (*n* = 13)	20% (*n* = 6)	*0.22*
Outcomes of TRENDS			
Total wear days (days)	61.7 ± 21.8 70.0 [6–91]	59.9 ± 27.8 67.5 [8–93]	*0.78*
Wear time per day (hour)	21.3 ± 4.9 23.0 [3.8–23.8]	20.0 ± 5.7 23.0 [1.1–24.0]	*0.36*
Response rate of health-related surveys (%)	88.7 ± 0.3 100.0 [9–100]	NA NA	
Any arrhythmic events	69% (*n* = 25)	73% (*n* = 22)	*0.72*
Manual action	8% (*n* = 3)	10% (*n* = 3)	*0.81*
Appropriate shock	3% (*n* = 1)	7% (*n* = 2)	*0.45*
Clinical outcomes			
Interruption due to skin trouble	3% (*n* = 1)	0% (*n* = 0)	*0.10*
HF hospitalization	7% (*n* = 2)	3% (*n* = 1)	*0.76*
ICD indication	50% (*n* = 18)	53% (*n* = 16)	*0.79*
ICD implantation	25% (*n* = 9)	43% (*n* = 13)	*0.15*
Medical intervention	7% (*n* = 2) *	NA	

* Direct oral anticoagulant(s) initiated because atrial fibrillation was documented. Beta-blocker dose increased because of a high heart rate. Abbreviations: Ht, height; Wt, weight; BSA, body surface area; IHD, ischemic heart disease; NIHD, non-ischemic heart disease; WCD, wearable cardioverter-defibrillator; ACS, acute coronary syndrome; HF, heart failure; HT, hypertension; HL, hyperlipidemia; DM, diabetes mellitus; ICD, implantable cardioverter-defibrillator.

**Table 2:** Changes in assessment parameters and medication use.

**Parameters** **TREND Group**	**Initial** ***n* = 36**	**Latest** ***n* = 34**	***p*-Value**
Physical examination			
Systolic BP (mmHg)	130.9 ± 32.6	119.0 ± 21.3	*0.4*
HR (bpm)	90.0 ± 35.8	67.5 ± 11.9	*0.03*
TTE			
LVEF (%)	33.5 ± 16.8	40.1 ± 13.7	*0.03*
LVDd (mm)	60.2 ± 9.8	55.3 ± 9.8	*0.003*
LVDs (mm)	49.5 ± 12.6	42.5 ± 10.5	*<0.001*
Blood labor			
BNP (pg/mL)	311.1 ± 385.2	151.4 ± 186.9	*0.1*
Cr (mg/dL)	1.0 ± 0.4	1.0 ± 0.2	*0.5*
eGFR (mL/min/1.73)	68.8 ± 25.9	61.9 ± 17.4	*0.1*
LDL (mg/dL)	111.1 ± 44.2	NA	NA
Hba1c (%)	6.3 ± 1.3	NA	NA
Medication use			
ACEi/ARB/ARNI	61% (*n* = 22)	88% (*n* = 31)	*0.003*
BB	33% (*n* = 12)	45% (*n* = 15)	*0.3*
MRA	58% (*n* = 21)	67% (*n* = 22)	*0.6*
SGLT2i	28% (*n* = 10)	26% (*n* = 9)	*0.6*
Diuretics	36% (*n* = 13)	44% (*n* = 15)	*0.3*
DOAC	28% (*n* = 10)	26% (*n* = 9)	*0.6*
Antiplatelet	14% (*n* = 5)	15% (*n* = 5)	*0.6*
**Historical Control**	**Initial** ***n* = 30**	**Latest** ***n* = 24**	***p*-Value**
Physical examination	122.7 ± 22.6	117.1 ± 17.5	*0.4*
Systolic BP (mmHg)	76.0 ± 15.2	69.6 ± 15.5	*0.* *2*
HR (bpm)	85.8 ± 24.5	69.3 ± 15.2	*0.0* *1*
TTE			
LVEF (%)	44.9 ± 16.0	45.8 ± 22.7	*0.* *9*
LVDd (mm)	56.0 ± 11.0	56.1 ± 11.0	*1.0*
LVDs (mm)	42.6 ± 16.0	41.7 ± 14.6	*0.8*
Blood labor			
BNP (pg/mL)	264.6 ± 285.9	171.9 ± 226.7	*0.* *2*
Cr (mg/dL)	0.9 ± 0.4	0.9 ± 0.3	*0.* *6*
eGFR (mL/min/1.73)	73.3 ± 27.6	66.2 ± 25.0	*0.* *4*
LDL (mg/dL)	111.1 ± 44.2	NA	NA
Hba1c (%)	6.3 ± 1.3	NA	NA
Medication use			
ACEi/ARB/ARNI	63% (*n* = 19)	58% (*n* = 14)	*0.7*
BB	67% (*n* = 20)	63% (*n* = 15)	*0.8*
MRA	50% (*n* = 15)	50% (*n* = 12)	*1.0*
SGLT2i	3% (*n* = 1)	8% (*n* = 2)	*0.4*
Diuretics	60% (*n* = 18)	58% (*n* = 14)	*0.* *9*
DOAC	37% (*n* = 11)	38% (*n* = 9)	*0.9*
Antiplatelet	30% (*n* = 9)	23% (*n* = 7)	*0.9*

Abbreviations: BP, blood pressure; HR, heart rate; TTE, transthoracic echocardiography; LVEF, left ventricular ejection fraction; LVDd, left ventricular diastolic dimension; LVDs, left ventricular systolic dimension; BNP, brain natriuretic peptide; Cr, creatinine; eGFR, estimated glomerular filtration rate; LDL, low-density lipoprotein; Hb, hemoglobin; ACEi, angiotensin-converting enzyme inhibitor; ARB, angiotensin receptor blocker; ARNI, angiotensin receptor neprilysin inhibitor; BB, beta-blocker; SGLT2i sodium-glucose cotransporter 2 inhibitor; DOAC, direct oral anticoagulant.

## Data Availability

The datasets generated and/or analyzed during the current study are not publicly available due to institutional and patient privacy regulations but are available from the corresponding author on reasonable request and with appropriate institutional approval.

## Abbreviations

The following abbreviations are used in this manuscript:

WCDs	Wearable cardioverter-defibrillators
RMS	Remote monitoring system
ICD	Implantable cardioverter-defibrillator
HF	Heart failure
LVEF	Left ventricular ejection fraction
NYHA	New York heart association
ICU	Intensive care unit
Ht	Height
Wt	Weight
BSA	Body surface area
IHD	Ischemic heart disease
NIHD	Non-ischemic heart disease
ACS	Acute coronary syndrome
HT	Hypertension
HL	Hyperlipidemia
DM	Diabetes mellitus
BP	Blood pressure
HR	Heart rate
TTE	Transthoracic echocardiography
LVDd	Left ventricular diastolic dimension
LVDs	Left ventricular systolic dimension
BNP	Brain natriuretic peptide
Cr	Creatinine
eGFR	Estimated glomerular filtration rate
LDL	Low-density lipoprotein
Hb	Hemoglobin
ACEi	Angiotensin-converting enzyme inhibitor
ARB	Angiotensin receptor blocker
ARNI	Angiotensin receptor neprilysin inhibitor
BB	Beta-blocker
SGLT2i	Sodium-glucose cotransporter 2 inhibitor
DOAC	Direct oral anticoagulant
